# Targeting Microbial Biofouling by Controlling Biofilm Formation and Dispersal Using Rhamnolipids on RO Membrane

**DOI:** 10.3390/membranes12100928

**Published:** 2022-09-25

**Authors:** Zahidullah, Muhammad Faisal Siddiqui, Shamas Tabraiz, Farhana Maqbool, Fazal Adnan, Ihsan Ullah, Muhammad Ajmal Shah, Waqar Azeem Jadoon, Tariq Mehmood, Sadia Qayyum, Ziaur Rahman

**Affiliations:** 1 Department of Microbiology, Hazara University, Mansehra 21300, Pakistan; 2 School of Natural and Applied Sciences, Canterbury Christ Church University, Canterbury CTI 1QU, UK; 3 Atta ur Rahman School of Applied Biosciences, National University of Sciences & Technology, Islamabad 44000, Pakistan; 4 Department of Biological Sciences, Faculty of Science, King Abdulaziz University, P.O. Box 80203, Jeddah 21589, Saudi Arabia; 5 Department of Pharmacy, Hazara University, Mansehra 21120, Pakistan; 6 Department of Earth and Environmental Sciences, Hazara University, Mansehra 21300, Pakistan; 7 Department of Bioinformatics, Hazara University, Mansehra 21300, Pakistan; 8 Department of Microbiology, Abdul Wali Khan University, Mardan 23200, Pakistan

**Keywords:** membrane biofouling, biofilm control, dispersal, rhamnolipids, biomass, EPS

## Abstract

Finding new biological ways to control biofouling of the membrane in reverse osmosis (RO) is an important substitute for synthetic chemicals in the water industry. Here, the study was focused on the antimicrobial, biofilm formation, and biofilm dispersal potential of rhamnolipids (RLs) (biosurfactants). The MTT assay was also carried out to evaluate the effect of RLs on biofilm viability. Biofilm was qualitatively and quantitatively assessed by crystal violet assay, light microscopy, fluorescence microscopy (bacterial biomass (µm^2^), surface coverage (%)), and extracellular polymeric substances (EPSs). It was exhibited that RLs can reduce bacterial growth. The higher concentrations (≥100 mg/L) markedly reduced bacterial growth and biofilm formation, while RLs exhibited substantial dispersal effects (89.10% reduction) on preformed biofilms. Further, RLs exhibited 79.24% biomass reduction while polysaccharide was reduced to 60.55 µg/mL (*p* < 0.05) and protein to 4.67 µg/mL (*p* < 0.05). Light microscopy revealed biofilm reduction, which was confirmed using fluorescence microscopy. Microscopic images were processed with BioImageL software. It was revealed that biomass surface coverage was reduced to 1.1% at 1000 mg/L of RLs and that 43,245 µm^2^ of biomass was present for control, while biomass was reduced to 493 µm^2^ at 1000 mg/L of RLs. Thus, these data suggest that RLs have antimicrobial, biofilm control, and dispersal potential against membrane biofouling.

## 1. Introduction

Membrane technologies play an important role in the treatment of seawater and wastewater [1]. However, bacterial biofilms on the membrane pose a major problem for water purification systems based on the membrane [2]. Membrane biofouling is the attachment and growth of microbial cells on the membrane surface and its pores. Membrane biofouling reduces the membrane efficiency, which results in economic losses and technological challenges [3].

Treatment or disinfection of these waters for commercial use does not establish sterile conditions over a long period. Membranes with antibacterial activities were also tested [4]. Thus, if a small amount of nutrients is available, the remaining bacteria will multiply and lead to membrane biofouling. Therefore, biofouling is a serious problem [3].

It was exhibited that conventional treatment methods are very expensive; many chemicals are not stable, and some showed toxicity effects. These limitations can be overcome by rhamnolipids (biosurfactants) as they have benefits compared to synthetic chemicals due to stability, low cost, and less toxic effects [5]. Biosurfactants are mostly used as cleaning chemicals due to their property of being surface-acting agents which solubilize or remove the attached biomass on the surfaces [6]. The most investigated and studied surfactants are from *Pseudomonas aeruginosa* and are biologically produced [5].

Hence, the current study was focused on mitigating membrane biofouling by targeting membrane biofilm formation, biofilm dispersal, and EPS reduction using different concentrations of rhamnolipids.

## 2. Materials and Methods

### 2.1. Chemicals, Accessories, and Microbes

All chemicals were purchased from Sigma Aldrich, Deajunge, Korea, Alfa Aesar (Haverhill, MA, USA). Rhamnolipids were purchased from AGAE Technologies (Corvallis, OR, USA). Glassware and accessories were purchased from MUSAJI Adam and Sons (Abbottabad, Pakistan. The nutrient broth medium was prepared according to the protocol in [7], and this medium was subjected to sterilization at 121 °C for 15 min. Bacterial mixtures (multispecies) were tested for biofilm and biofouling studies. The mixed culture was isolated from the reverse osmosis (RO) plant in Mansehra. Coupons for reverse osmosis (RO) membranes (Model TW30-1812-100HR, Dow FilmTec Membranes (USA) and polystyrene microplates (24 wells) were provided by local suppliers.

### 2.2. Biofouled RO Membrane Sample and Bacteria Isolation

Biofouled membrane samples were collected from the RO membrane filtration plant situated in Mansehra, Pakistan. To isolate bacteria, the samples were collected in sterilized 50 mL Falcon tubes and analyzed within an hour. Biofilm samples were scraped off the membrane surface into phosphate-buffered saline (PBS) and then sonicated for 1 min in a sonicator and centrifuged for 1 min at 3000 rpm to remove debris. The supernatant was centrifuged again for 5 min at 4500 rpm. The pellet was resuspended in 15 mL saline and used for bacterial isolation. Bacteria samples were also grown in nutrient broth medium at 200 rpm for 48 h at 25 °C under shaking conditions in a shaking incubator and were stored in glycerol at −80 °C for further use.

### 2.3. Bacterial Growth Assays

The initial step of testing rhamnolipids was started from their effects on bacterial growth. For this purpose, bacteria were inoculated in the nutrient broth (NB). Bacterial samples were incubated at 25 °C under continuous shaking conditions (200 rpm) for 24 h. Desired bacterial cultures (OD of 0.001) were added to the microtiter plate. Then, 50 μL of RLs (1–800 mg/L) was added from the stock solution to each well, and plates were incubated at 25 °C under shaking conditions for 24 h. A control was also included in parallel; however, for the control (nontreated), rhamnolipids were not added but an equal amount of sterilized distilled water was added. All experiments were performed in triplicate [8].

### 2.4. Microtiter Assays for Biofilm Control

As stated previously, bacterial cells were cultured. Various concentrations (1–800 mg/L) of RLs were applied to each well in the plates. The biofilms were then formed on the surface of polystyrene (24-well) plates in a shaking incubator for 24 h at 30 °C and 120 rpm. In the next step, after 24 h, the liquid broth was removed from the plates, and the plates were rinsed with sterilized distilled water. This step was carried out very carefully to avoid biofilm removal. Then, 1 mL ethanol (99.9%) was added to each well for the fixation of biofilm in each well. It was incubated for 10 min at room temperature for the fixation of biofilm. Then, ethanol was removed, and plates were air-dried. A stain solution (0.1%) was prepared from crystal violet dye, containing 0.1 g of dye in 100 mL distilled water. For staining, 1 mL of dye solution was added to all wells, and it was stained for 15 min. Then, all wells were rinsed with distilled water to remove the nonattached extra stain. Then, glacial acetic acid (33% volume/volume) solution was prepared by mixing 33 mL of glacial acetic acid with 67 mL of distilled water. Then, 1 mL of the glacial acetic acid solution was added to each well to dissolve the bound dye attached to the biofilms. Additionally, a spectrophotometer was used to measure the optical density of the sample in a plastic cuvette at 595 nm [9]. Experiments were performed in triplicate.

### 2.5. MTT Biofilm Viability Assay

Bacterial viability withing biofilms was determined using the MTT (3-(4,5-dimethylthiazol-2yl)-2,5-diphenyltetrazolium bromide) assay [10].

### 2.6. Biofilm Dispersal in Microtiter Plates by RLs

RLs were used to study biofilm dispersion in preformed (24 h) biofilms. Biofilms were produced on 24-well microtiter plates in a nutrient medium (as mentioned above). Preformed biofilms were gently rinsed with sterilized water. PBS, along with appropriate quantities of RLs, was poured into a series of wells to test the efficacy of RLs. Simultaneously, a control without RLs was also included in the study. All plates were incubated for 2, 4, and 24 h at 30 °C in an incubator, on a rotating platform shaker with shaking of 100 rpm [11]. The biofilm was measured using the method described in Section 2.6 after incubation.

### 2.7. Biofilm Dispersal on RO Membrane

Bacteria were cultivated in nutrient broth under shaking conditions at 120 rpm. Bacterial cells were diluted to desired concentrations (0.001 OD). Bacterial cultures were vortexed and added to sterilized 50 mL tubes (total volume was kept at 40 mL per tube). Bacterial cells in a Falcon tube (50 mL) were allowed to form biofilms on RO membrane coupons attached on glass slides for 24 h at 120 rpm and 30 °C. After 24 h, the media was removed and the biofilm on the membrane was gently washed with sterilized water. Then, RLs in various concentrations were added to all tubes including sterilized distilled water, and tubes were treated for 2 h. In the control, only distilled water was added instead of RLs. After 2 h of treatment, the RO membrane was subjected to EPS and biomass analysis.

### 2.8. EPS and Biomass Extraction

After 2 h of treatment, EPSs and biomass were extracted from the membrane’s surface. A cell scraper (Cell scrapers have long handles and beveled, angled heads to harvest cells from different cell and tissue culture vessels. These products also reduce cell damage and provide even contact.) was used to remove the biofilm physically from the membrane’s surface, which was then dissolved in 20 mL of sterilized water and vortexed for one minute in tubes. The membrane was cut into suitable pieces using scissors and scraped on the surface with tweezers. Small sections of the membrane were placed in the same tube and vortexed for four minutes. The samples were then centrifuged in a centrifuge apparatus at 10,000 rpm for 15 min at 4 °C. Following centrifugation, the supernatant, known as extracellular polymeric substances (EPSs), was transferred to a separate tube, while the residual pellet was referred to as biomass [11].

### 2.9. Measurement of Cell Biomass Concentration

Pellets were rinsed with sterilized water, and 10 mL of sterilized water was added to each tube containing the pellet. After that, vortexing was performed to thoroughly mix the pellets in the sterilized water. Using a spectrophotometer, the OD of the sample (biomass) was measured at 600 nm.

### 2.10. Polysaccharide Quantification

The supernatant was considered as soluble EPSs, and 1 mL was taken from the supernatant and poured into a labeled glass tube. Then, 0.5 mL of 5% phenol was added to the tube. About 2.5 mL of concentrated H_2_SO_4_ solution was added carefully to the mixture. The mixture was incubated for 10 min at room temperature, and absorbance was measured spectrophotometrically at 492 nm [12].

### 2.11. Protein Quantification

The Bradford assay was used for protein quantification [13].

### 2.12. Microscopic Visualization of Biofilm

The RLs’ ability to disperse biofilm development was also verified microscopically. In brief, mixed bacteria were allowed to form biofilms on a membrane attached to glass slides (1.8 cm in diameter and 7.3 cm in length), which were placed in 50 mL Falcon tubes containing nutrient medium and incubated for 24 h. Following incubation, various quantities of RLs (control, 100 mg/L, 400 mg/L, 800 mg/L, and 1000 mg/L) were added and incubated for another 2 h at room temperature. The membrane coupons were then stained for 20 min at room temperature using crystal violet dye (0.1% *w*/*v*). Light microscopy at a magnification of 40 objective was performed to examine stained membranes containing biofilms [14].

Biofilm samples on the RO membrane were further analyzed by fluorescence microscopy. After incubation, biofilm samples were washed gently with saline water, and 0.1% fluorescein isothiocyanate (FITC) was used to stain the biofilm, which was then kept in the dark at room temperature for 15 min. To remove the unbound stain, the slides were washed with sterilized distilled water. Then, stained slides were subjected to fluorescence microscopy using a fluorescence microscope. The stained biofilms were visualized, and images were captured at 488 nm excitation and 530 nm emission. Digital images were viewed using NIS-AR Element Software (Nikon, Tokyo, Japan). Images were processed using image analysis software BioImageL v.2.1 (developed by Dr. Luis Chávez de Paz). Percent surface coverage and biomass (µm^2^) were calculated from images using BioImageL (Luis E. Chávez de Paz, Department of Oral Biology, Malmö University, Malmö 20506, Sweden).

### 2.13. Statistical Analysis

All experiments were carried out in triplicate. The average and standard error were calculated using Microsoft Excel, and standard error was presented in the form of error bars in the graphs using Microsoft Excel 2010 (Microsoft Corporation, Redmond, WA, USA).

## 3. Results and Discussion

### 3.1. Effect of Rhamnolipid Concentrations on Bacterial Growth

**Figure 1:**
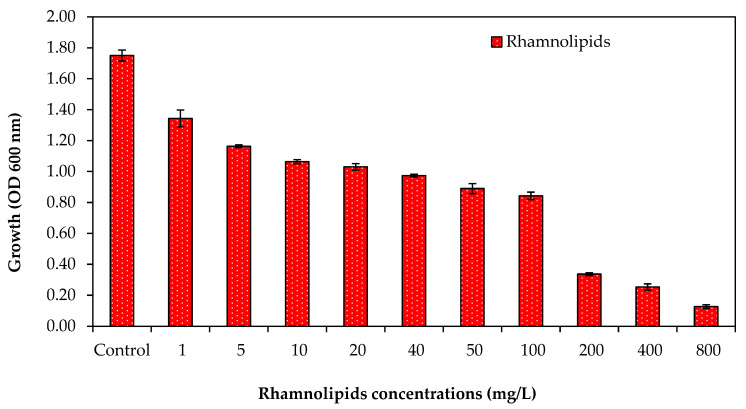
Growth inhibition test for RO bacterial mixture in the presence of different concentrations of rhamnolipids. Error bars are SDs (*n* = 3).

It was found that at low RL concentrations, a minor reduction in bacterial growth was detected, as shown in Figure 1. Bacterial growth, in the presence of RLs at 5 mg/L, showed 1.16 OD. The results indicated that RLs inhibited bacterial growth, and it was further noted that an increase in growth reduction was observed with an increase in the concentration of RLs, as shown in Figure 1; the OD of 0.84 was observed in the presence of RLs at concentrations up to 100 mg/L. However, increased RL concentrations over 100 mg/L drastically reduced bacterial growth. Percent growth reduction was also calculated for the effect of different concentrations of RLs against the RO bacterial mixture. At lower RL concentrations (5–50 mg/L), less than 50% growth reduction was observed (Table 1). These results suggest that RLs can inhibit bacterial growth.

### 3.2. Effect of Rhamnolipid Concentrations on the Growth of Bacteria

**Figure 2:**
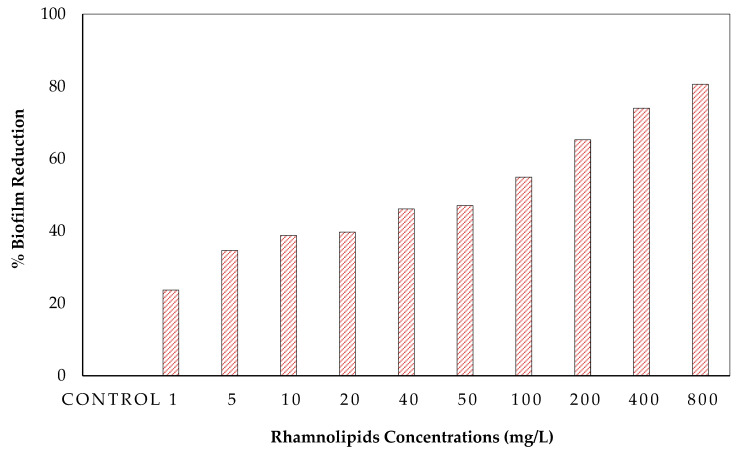
Effect of different concentrations of rhamnolipids on percent biofilm reduction.

Rhamnolipid concentrations were tested against the biofilm of the RO bacterial consortium (Figure 2). It was found that RLs resulted in a biofilm reduction of 34.63% when 5 mg/L of rhamnolipids was used in plates (Figure 2). Furthermore, increasing the RL concentration from 20 to 800 mg/L resulted in biofilm reduction of up to 39.72–80.61% (Figure 2). Most of the RL concentrations used to inhibit biofilm development against the RO bacterial mixture revealed biofilm prevention. These results showed that RLs can control the biofilm formation caused by RO multispecies. At low concentrations, the effect was not that great, but when the concentrations were increased, a strong effect on biofilm formation control was shown. Results were also compared with other studies. Do Valle Gomes and Nitschke [15] studied surfactin as a biosurfactant for biofilm control. They found that it has anti-mycoplasma and antibacterial activities. Furthermore, RLs regulate bacterial cell viability, allowing biofilm adherence and detachment on many surfaces by forming holes in the inner structure of biofilm [16].

### 3.3. MTT Assay for Cell Viability in Biofilms upon Using RLs

**Figure 3:**
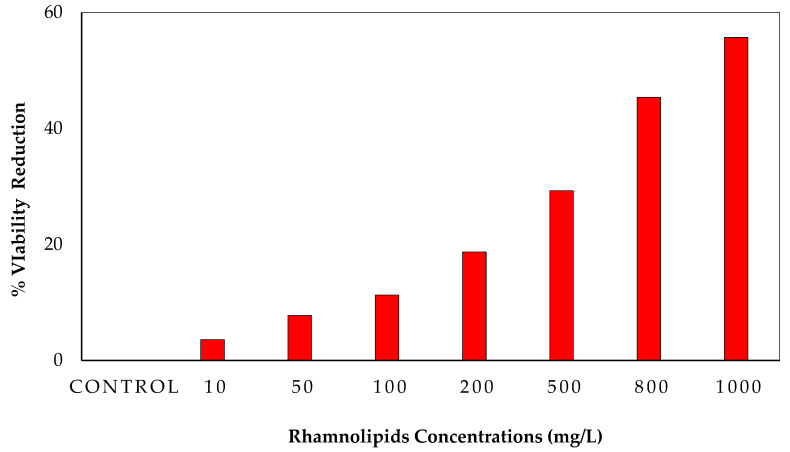
Viability reduction of biofilm formation in the presence of different concentrations of rhamnolipids against the bacterial consortium.

The purpose of the MTT assay was to determine the viability of cells in biofilms. This experiment employed a range of RL concentrations (control, 10, 50, 100, 200, 400, 800, and 1000 mg/L). Figure 3 shows that RLs can kill 55.682% of bacterial viable cells at 1000 mg/L. Bacterial viability in biofilms has previously been studied using the MTT assay [17]. Another advantage of the MTT assay is that it has been used in the past to evaluate the antibacterial agents’ effect on strains of clinical microbial consortia [18]. In our study, a microbial viability reduction of 52% was observed in the MTT assay, which means RLs have antimicrobial activity against bacteria that kills more than 50% of bacteria.

### 3.4. Effect of Rhamnolipids on Biofilm Dispersal

**Figure 4:**
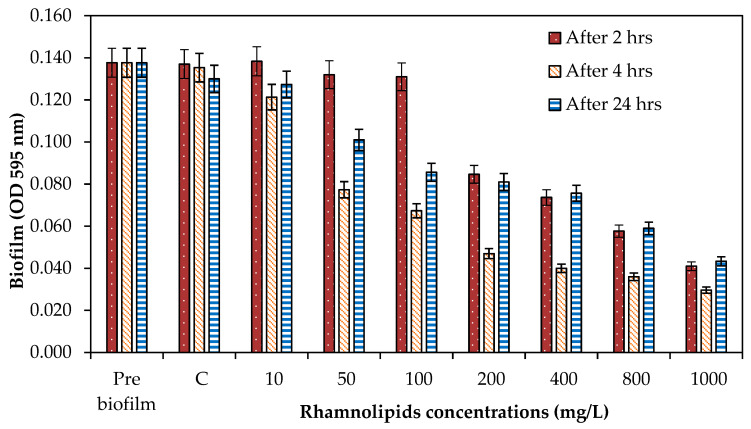
The effect of various RL concentrations on the dispersal of a 24 h old biofilm on a polystyrene surface across a range of exposure times (2, 4, 24 h). Error bars are SDs (*n* = 3).

**Figure 5:**
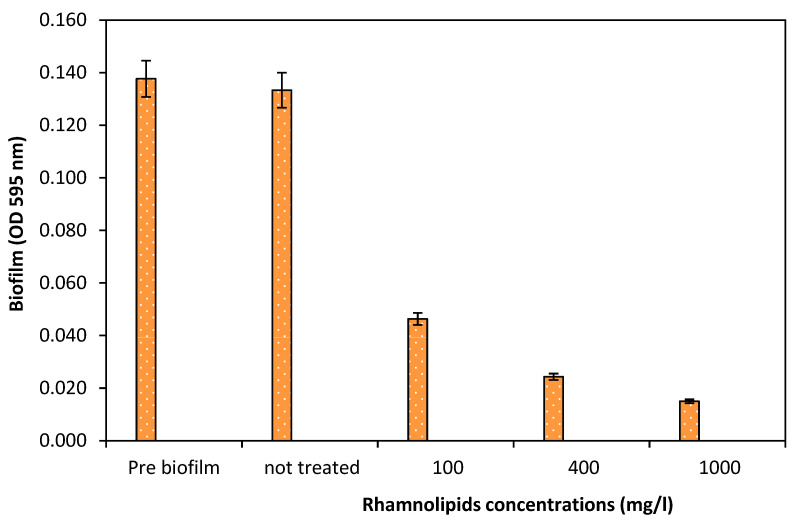
The effect of various rhamnolipid concentrations on the dispersal of a 24 h old biofilm on a RO membrane (2 h). Error bars are SDs (*n* = 3).

After biofilm development, the biofilm dispersion capacity of RLs was tested on a preformed (24 h old) biofilm. As illustrated in Figure 4, it was found that there was no great removal at 10 mg/L of RLs; however, the dispersal efficacy was increased when the concentration of RLs was increased. It was exhibited that when the exposure period was increased to 24 h, the dispersal efficacy was reduced compared to 2 and 4 h of exposure (Figure 4). Furthermore, the biofilm removal potential of RLs (100, 400, and 1000 mg/L) was tested on the membrane surface for 2 h of exposure. Rhamnolipids exhibited a 66.34% reduction at 100 mg/L, while 89.10% biofilm reduction was achieved at 1000 mg/L (Figure 5). Wood et al. [19] revealed that PA14 supernatants effectively disperse *D. vulgaris* biofilms and that the biological substrate for this dispersion is the presence of rhamnolipids. PA14 supernatants dispersed biofilms more effectively than protease and were capable of dispersing biofilms from *D. desulfuricans*, *E. coli* MG1655, and *S. aureus*. Previously, it was discovered that *P. aeruginosa* RLs and the QS signal 3oxoC12HSL disperse *E. coli* biofilms in a synergetic manner (Cohen and Exerowa, 2007). According to Wood et al. [19], it is possible to disperse industrially significant biofilms using supernatants containing RLs.

### 3.5. Effect of Rhamnolipids on EPS Production

**Figure 6:**
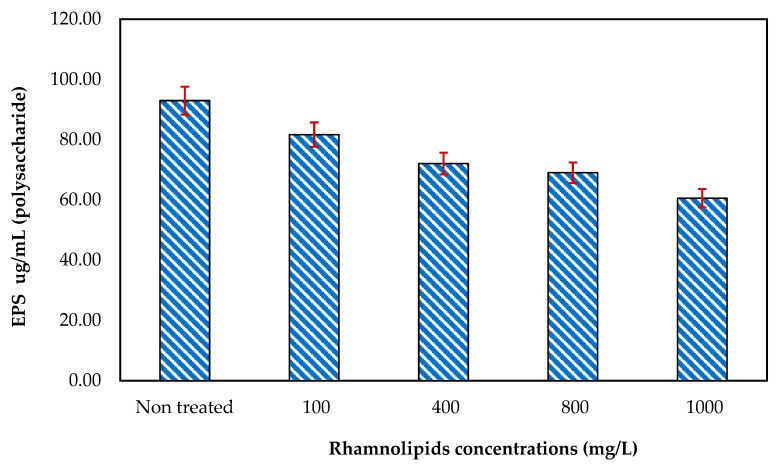
The effect of rhamnolipids (100–1000 mg/L) on the EPS (polysaccharide) synthesis on the RO membrane. Error bars are SDs (*n* = 3).

**Figure 7:**
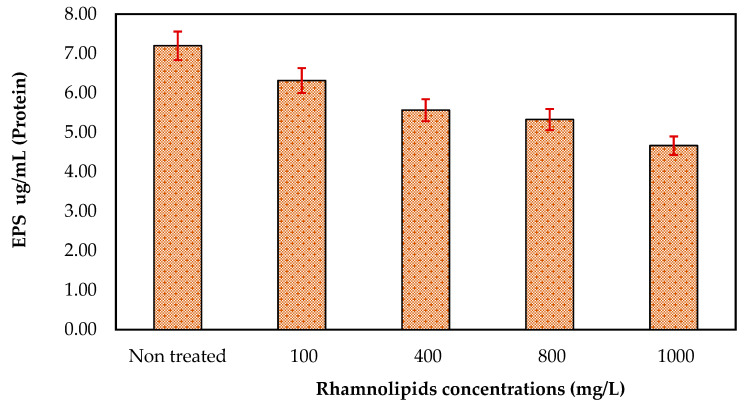
The effect of rhamnolipids (100–1000 mg/L) on the EPS (protein) synthesis on RO membrane. Error bars are SDs (*n* = 3).

RLs were tested at different concentrations (ranging from 100 to 1000 mg/mL) to determine their ability to remove EPSs (polysaccharides and proteins). According to the results of the current experiment, at 1000 mg/mL, RLs have the strongest effects on the removal of the extracellular polysaccharides of mixed bacteria, as seen in Figure 6. There was 92.94 µg/mL of polysaccharides present in control on the membrane surface, while the polysaccharides were reduced to 60.55 µg/mL at an RL concentration of 1000 mg/mL. These results revealed that rhamnolipids can remove polysaccharides from the membrane surface. Effects of RLs on the removal of EPSs (proteins) (Figure 7) were also studied using different concentrations. Although at initial concentrations rhamnolipids showed less effect (protein 6.32 µg/mL), at higher concentrations, i.e., 1000 mg/L, the rhamnolipids reduced the protein concentration to 4.67 µg/mL. These results further indicated that rhamnolipids can also remove extracellular proteins in pre-established biofilm on membrane surfaces. In one of the studies by Xiong and Liu [20], it was found that bacterial EPSs promote bacterial adhesion to solid surfaces such as membranes. EPSs have been suggested to be irreversible foulants of membrane fouling due to their ability to reduce bacteria’s susceptibility to antibiotics and so function as a shelter for bacteria inside the biofilm–EPS matrix [21]. However, certain enzymes can also hydrolyze EPSs, suggesting a potential way to limit EPS-mediated microbial adhesion and biofouling of membranes since extracellular proteins and polysaccharides constitute most of the EPSs produced by bacteria.

### 3.6. Effect of Rhamnolipids on Biomass

**Figure 8:**
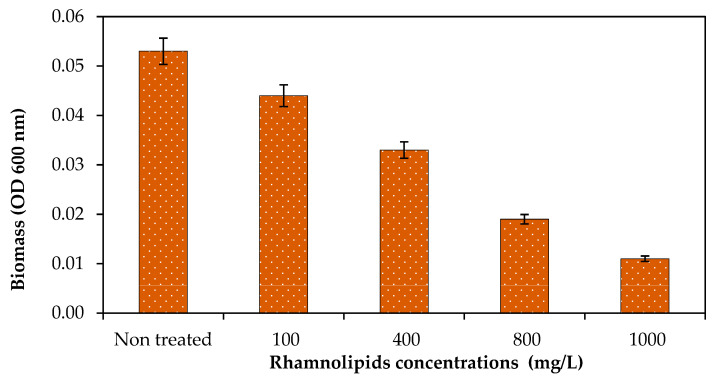
The effect of rhamnolipids (100–1000 mg/L) on the bacterial biomass on the RO membrane. Error bars are SDs (*n* = 3).

The purpose of the study was to see how RLs can affect mixed bacteria cell biomass on the membrane surface. Pre-established biofilm on the membrane surface was exposed to different concentrations of RLs. Biofilm biomass dispersal was studied using different concentrations of RLs ranging from 100 to 1000 g/mL, as shown in Figure 8. It was found that RLs at 100 mg/L reduced 16.98% biomass on the membrane surface, while it was observed that at 1000 mg/L, 79.24% biofilm biomass was reduced on the membrane surface. As shown in Figure 8, RLs at a concentration of 1000 g/mL effectively reduced cell biomass. Thus, it was exhibited that RLs can also disperse biofilm on the membrane surface. Siddiqui et al. [7] studied biomass reduction on membrane coupons.

### 3.7. Microscopic Visualization of Biofilms

**Figure 9:**
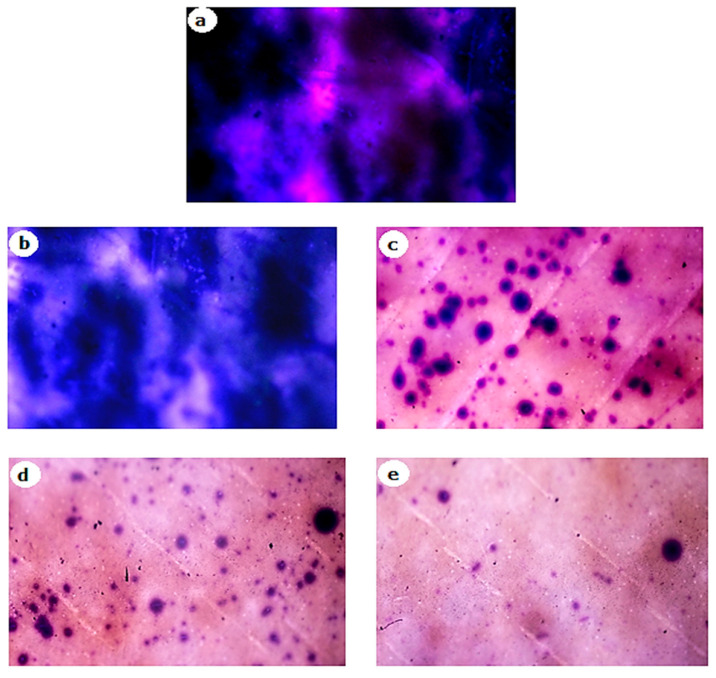
Light microscopic images of the effect of different concentrations of rhamnolipids on biofilm dispersal on RO membrane. (**a**) Control, (**b**) 100 mg/L, (**c**) 400 mg/L, (**d**) 800 mg/L, (**e**) 1000 mg/L.

**Figure 10:**
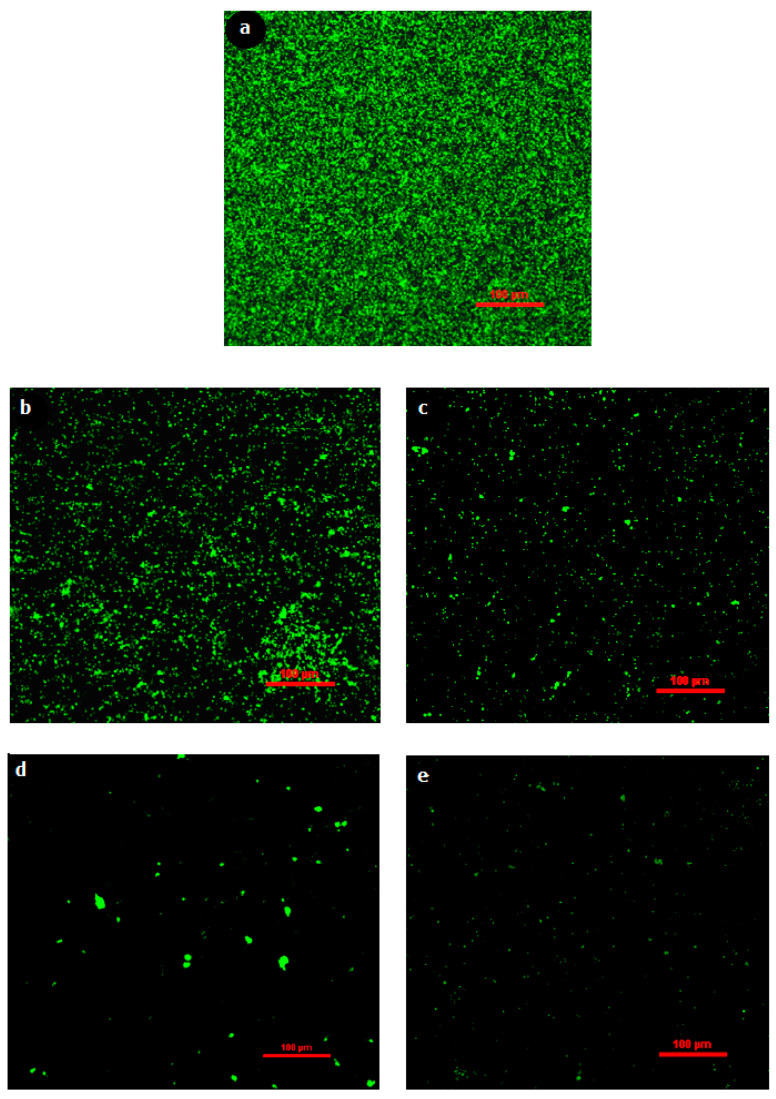
Fluorescence microscopic visualization of the effect of different concentrations of rhamnolipids on biofilm dispersal on RO membrane. (**a**) Control, (**b**) 100 mg/L, (**c**) 400 mg/L, (**d**) 800 mg/L, (**e**) 1000 mg/L.

To further confirm the results, biomass removal was also studied under light and fluorescence microscopes. After 2 h of exposure of biofilms on membrane surface to different concentrations of RLs and an incubation period, stained membrane pieces were placed on glass slides with the biofilm facing up and examined under a light microscope at 400× *g* magnification. Figure 9 shows that RLs at 100 mg/L slightly dispersed biofilm from the membrane surface, while at 1000 mg/L, the biofilm dispersal was greatly increased. Light microscopy revealed biofilm reduction, which was confirmed using fluorescence microscopy (Figure 10). Fluorescence microscopic images were processed with BioImageL software. It was revealed that biomass surface coverage was reduced to 1.1% at 1000 mg/L of RLs and that 43,245 µm^2^ biomass was present for control, while the biomass was reduced to 493 µm^2^ at 1000 mg/L of RLs (Table 2). In one of the studies performed by Al-Juboori and Yusaf [22], it was exhibited that light microscopy is an important technique for examining the presence of biofilm on surfaces. Light microscopy has an estimated resolution of about 1 µm [23]. This sort of microscopy is often used for early biofilm examination since it shows the presence of biomass on surfaces [24]. When using light microscopy, it is difficult to obtain information on the depth and spatial distribution of the biofilm, and the resolution of the resulting biofilm image is inadequate. This might have an impact on accuracy since the wet biomass has a refraction index that is affected by the light microscope [24].

## 4. Conclusions

It was exhibited that RLs possess anti-microbial activity against an RO bacterial consortium. It was observed that RLs have the ability to greatly inhibit bacterial growth at higher RL concentrations. Furthermore, it was revealed that RLs can mitigate biofilm formation from the start on a polystyrene surface. Moreover, the MTT assay showed that RLs can reduce bacterial viability in biofilms. It was observed that with increasing RL concentration, the cell viability was greatly reduced in biofilms. In addition, it was found that RLs can disperse an already established biofilm on a membrane surface, which exhibited the biofilm dispersal potential of RLs. Moreover, biofilm dispersal on the membrane surface was further confirmed by biomass analysis and microscopy. Biofouling mitigation was also correlated with the reduction in EPSs (proteins and polysaccharides) on the RO membrane surface.

## Figures and Tables

**Table 1:** Effect of different concentrations of rhamnolipids on bacterial growth reduction.

Conc. mg/L	Absorbance	% Reduction
Control	1.750	0.000
1	1.343	23.253
5	1.163	33.536
10	1.063	39.250
20	1.030	41.154
40	0.973	44.392
50	0.890	49.153
100	0.843	51.819
200	0.337	80.766
400	0.253	85.527
800	0.127	92.763

**Table 2:** Effect of RA on biofilm surface coverage and biomass reduction.

Sample (mg/L)	Surface Coverage (%)	Biomass (µm^2^)	% Biomass Reduction
Control	96.0%	43,245	00.00
100	21.7%	9766	77.41
400	4.8%	2159	95.00
800	1.3%	567	98.68
1000	1.1%	493	98.95
